# NUSAP1 Could be a Potential Target for Preventing NAFLD Progression to Liver Cancer

**DOI:** 10.3389/fphar.2022.823140

**Published:** 2022-04-01

**Authors:** Taofei Zeng, Guanglei Chen, Xinbo Qiao, Hui Chen, Lisha Sun, Qingtian Ma, Na Li, Junqi Wang, Chaoliu Dai, Feng Xu

**Affiliations:** ^1^ Department of General Surgery, Hepatobiliary and Splenic Surgery Ward, Shengjing Hospital of China Medical University, Shenyang, China; ^2^ Department of Oncology, Shengjing Hospital of China Medical University, Shenyang, China; ^3^ Department of General Surgery, Pancreatic and Thyroid Surgery Ward, Shengjing Hospital of China Medical University, Shenyang, China; ^4^ Department of Pediatrics, Shengjing Hospital of China Medical University, Shenyang, China; ^5^ Department of Pediatrics, The Second Affiliated Hospital of DaLian Medical University, Dalian, China

**Keywords:** non-alcoholic fatty liver disease, liver fibrosis, hepatocellular carcinoma, bioinformatics analysis, drug targets

## Abstract

**Background:** Non-alcoholic fatty liver disease (NAFLD) has gradually emerged as the most prevalent cause of chronic liver diseases. However, specific changes during the progression of NAFLD from non-fibrosis to advanced fibrosis and then hepatocellular carcinoma (HCC) are unresolved. Here, we firstly identify the key gene linking NAFLD fibrosis and HCC through analysis and experimental verification.

**Methods:** Two GEO datasets (GSE89632, GSE49541) were performed for identifying differentially expressed genes (DEGs) associated with NAFLD progression from non-fibrosis to early fibrosis and eventually to advanced fibrosis. Subsequently, Gene Ontology (GO), Kyoto Encyclopedia of Genes and Genomes (KEGG) pathways enrichment analysis, protein-protein interaction (PPI) network were integrated to explore the potential function of the DEGs and hub genes. The expression of NUSAP1 was confirmed *in vivo* and *in vitro* NAFLD models at mRNA and protein level. Then, cell proliferation and migration under high fat conditions were verified by cell counting kit-8 (CCK-8) and wound-healing assays. The lipid content was measured with Oil Red O staining. Finally, the analysis of clinical survival curves was performed to reveal the prognostic value of the crucial genes among HCC patients via the online web-tool GEPIA2 and KM plotter.

**Results:** 5510 DEGs associated with non-fibrosis NAFLD, 3913 DEGs about NAFLD fibrosis, and 739 DEGs related to NAFLD progression from mild fibrosis to advanced fibrosis were identified. Then, a total of 112 common DEGs were found. The result of enrichment analyses suggested that common DEGs were strongly associated with the glucocorticoid receptor pathway, regulation of transmembrane transporter activity, peroxisome, and proteoglycan biosynthetic process. Six genes, including KIAA0101, NUSAP1, UHRF1, RAD51AP1, KIF22, and ZWINT, were identified as crucial candidate genes via the PPI network. The expression of NUSAP1 was validated highly expressed *in vitro* and vivo NAFLD models at mRNA and protein level. NUSAP1 silence could inhibit the ability of cell proliferation, migration and lipid accumulation *in vitro*. Finally, we also found that NUSAP1 was significantly up-regulated at transcriptional and protein levels, and associated with poor survival and advanced tumor stage among HCC patients.

**Conclusion:** NUSAP1 may be a potential therapeutic target for preventing NAFLD progression to liver cancer.

## Introduction

Nonalcoholic fatty liver disease (NAFLD) is one of the most widespread chronic liver diseases globally, affecting 25% of the general population and 85%–98% of morbidly obese patients worldwide (Friedman et al., 2018; Berardo et al., 2020). The disease progression of NAFLD is a dynamic process. In the early stage of NAFLD, hepatocytes begin to experience lipid deposition. As lipid deposition increases, lipotoxicity damages of hepatocytes aggravate, which in turn induces oxidative stress, leads to damage to cell mitochondria, triggers inflammatory factors release, and exacerbates hepatocyte damage (Schuster et al., 2018). With the aggravation of lipid deposition in hepatocytes and the occurrence of inflammation, about 20% of NAFLD patients can progress to non-alcoholic steatohepatitis (NASH) (Eslam et al., 2018; Younossi et al., 2018). Some patients with NASH progressively developed liver cirrhosis, hepatocellular carcinoma (HCC), and eventually liver disease-related death (Anstee et al., 2019). Therefore, the increasing prevalence of NAFLD worldwide is entirely worthy of clinical attention.

Until now, no specific pharmacological therapies are attainable for the treatment of NAFLD (Rinella and Sanyal, 2016). The physical therapy treatments for patients suffering from NAFLD and NASH was recommended to change lifestyle and balance nutrition, in addition to control weight loss and physical exercise. Contrary to expectations, the majority of patients fail to lose weight (Vilar-Gomez et al., 2015; Marchesini et al., 2016). Therefore, it is worth elucidating the intrinsic molecular pathogenesis of NAFLD, and more exhaustive and detailed research is needed to explore the molecular mechanism and obtain new specific therapeutic targets.

We conducted a more comprehensive bioinformatics analysis between normal liver tissues and non-fibrosis/fibrosis NAFLD tissues, advanced fibrosis and mild fibrosis NAFLD tissues in the present study. Based on comprehensive bioinformatics analyses, hub genes identification, biological processes and pathways were also determined. Finally, we also demonstrated that these genes were highly expressed in NAFLD-associated HCC patients and strongly associated with poor prognosis and more advanced tumor stages. Our realization will provide meaningful clues for the treatment of NAFLD and NAFLD-associated HCC.

## Methods

### Acquisition of Datasets

Three NAFLD-related datasets GSE89632 (Arendt et al., 2015), GSE49541 (Moylan et al., 2014) and GSE164441 (Yang et al., 2021) were downloaded from the Gene Expression Omnibus (GEO) database (https://www.ncbi.nlm.nih.gov/geo/) including the gene expression profile data and related annotation file. The search keywords used were “Nonalcoholic fatty liver disease” AND “*Homo sapiens.*” The inclusion criteria for the dataset are as follows: (1) Each dataset included contained at least 10 samples; (2) Each sample contained pathological information; (3) The raw data or gene expression profiling from these datasets are accessible at the GEO.

The details of these datasets were shown in Table 1. The platform for GSE89632 was Illumina HumanHT-12 WG-DASL V4.0 R2 expression beadchip, for GSE49541 was Affymetrix Human Genome U133 Plus 2.0 Array (HG-U133_Plus_2), and for GSE164441 was GPL20301, Illumina HiSeq 4000 (*Homo sapiens*). It is important to note that samples with no specific pathologic information were not analyzed.

**TABLE 1:** Characteristics of the included microarray datasets.

GSE ID	Participants Included	Tissues	Analysis	Platform	Year
GSE89632	21 cases with no fibrosis, 18 cases with fibrosis and 11 healthy controls	Liver	Array	GPL14951	2016
GSE49541	40 cases with mild fibrosis and 32 cases with advanced fibrosis	Liver	Array	GPL570	2013
GSE164441	10 cases with (NAFLD)- associated HCC tumor (*n* = 10) and adjacent non-tumor liver tissues (*n* = 10)	Liver	RNA-seq	GPL20301	2021

### DEGs Identification

The “limma” software package in R software was used to screen out DEGs based on the cut-off criteria (Ritchie et al., 2015). The conditions of |log2 FC| ≥ 0.5, adjust p-value < 0.05 (corrected by B and H method) was used for NAFLD without fibrosis *vs*. healthy liver sample and NAFLD with fibrosis *vs*. healthy liver sample group to obtain DEGs associated with NAFLD fibrosis. GSE49541 included 40 mild NAFLD liver tissue samples and 32 advanced NAFLD liver tissue samples. The cut-off criterion of adjust *p*-value < 0.05 (corrected by B and H method) was used to obtain DEGs associated with NAFLD fibrosis progression. The heatmap for the DEGs was created using the “ggplot2” package (https://ggplot2.tidyverse.org), and the Venn plot for co-expressed DEGs was integrated using the “ggvenn” (https://github.com/yanlinlin82/ggvenn) packages, and utilized for the following analysis.

### Functional and Pathway Enrichment Analysis

To investigate the functional biological roles of the co-expressed DEGs, Gene Ontology (GO) terms and Kyoto Encyclopedia of Genes and Genomes (KEGG) pathways were automatically completed and visualized by the Metascape (Zhou et al., 2019). p-value < 0.05 was set as the significance threshold.

### Protein-Protein Interaction Network Construction

The common significant DEGs were mapped to the STRING database (http://www. string-db. org/), then set confidence >0.4 to conduct the PPI network analysis (Szklarczyk et al., 2019), which were visualized by Cytoscape software (v3.8.1) (Shannon et al., 2003). Module analysis was performed to identify significant modules and explore the hub genes using the Molecular Complex Detection (MCODE V2.0.0) (Bader and Hogue, 2003), which is a plug-in of Cytoscape (MCODE score ≥3.5).

### Expression Levels and Prognostic Value of Hub Genes in HCC

First, we downloaded the gene expression matrix from GSE164441, composed of 10 cases with (NAFLD)-associated HCC tumor and paired adjacent non-tumor liver tissues. The “ggolot2” package visualized the expression of key genes in NAFLD-induced tumor tissues and adjacent non-tumor liver tissues. Clinical survival analyses were retrieved online using the online tools, Gene Expression Profiling Interactive Analysis (GEPIA2) (http://gepia.cancer-pku.cn/) and Kaplan-Meier Plotter (http://kmplot.com/analysis/). p-value = 0.05 and |log2FC| = 1 and were defined as the threshold for statistical significance (Tang et al., 2017). Finally, the HPA database (https://www.proteinatlas.org/) provides tissue and cellular distribution information of various human proteins using immunoassay technology (Uhlén et al., 2005; Berglund et al., 2008; Pontén et al., 2008; Uhlén et al., 2015; Thul et al., 2017; Uhlen et al., 2017).

### Cell Culture and Treatment

The human hepatocyte cell lines HL-7702 and hepatoma cell line MHCC-97H (iCell Bioscience Inc., Shanghai, China) were cultured in a humidified incubator at 37 °C with 5% CO2 using RPMI-1640 with 10% FBS and 1% P/S solution (Procell). To establish the *in vitro* NAFLD cell model, HL-7702 and MHCC-97H cells were cultured in the presence or absence of 1 mmol/L free fatty acids (FFA, containing oleic acid and palmitic acid at a 2:1 volume ratio) for 24 h and then used for the indicated assays (Long et al., 2019).

### Construction of NAFLD Model Mice

Ten male C57/BL6 mice (7–8 weeks old) were randomly divided into two groups, one was administrated with a 60% high-fat diet (HFD) (New Brunswick, NJ08901, United States) (*n* = 5) and the other with a control diet (CD, 10% fat) for 12 weeks (*n* = 5) (Jiang et al., 2019). The animals were housed in a temperature-controlled environment, with a 12-h light/dark cycle and received food and water ad libitum, at the center of experimental animals of Benxi Laboratory, Shengjing Hospital of China Medical University. The animal experiments received ethical clearance from the Ethics Committee of Shengjing Hospital of China Medical University (2021PS570K).

### Sample Collection

After 12 weeks’ treatment, the mice were sacrificed under deeply anesthesia. The livers were then perfused with PBS via the portal vein to remove the blood and divided into two pieces. One was preserved in 10% neutral-buffered formalin, while the other was immediately frozen in liquid N_2_ and stored at −80°C.

### RT-PCR

Trizol reagent (Invitrogen, CA, United States) was utilized to extract total RNA according to the manufacturer’s protocol. Total RNA was then reverse-transcribed into the complementary DNAs (cDNAs) with the PrimeScript RT Reagent Kit with gDNA Eraser (TaKaRa, Dalian, China) and amplified by GoTaq qPCR Master Mix (Promega, Madison, WI, United States) using the ABI ViiA 7 Real-time PCR system (Applied Biosystems, United States). The relative mRNA levels were normalized to those of the housekeeping gene GAPDH. All samples were determined by the 2^–∆∆CT^ method. The primer pairs used in this study are listed in Supplementary Table S1 **.**

### Cell Transfection

The NUSAP1 siRNA and negative controls were purchased from HANBIO (HANBIO, Shanghai, China). The transfection of Hl-7702 and MHCC-97H cells was conducted in accordance with manufacturer’s guidance.

### Cell Counting Kit-8 Assay

Cells transfected with si-NUSAP1 were plated in 96-well plates and cultured for 24 h. Then, the transfected cells were add with 10 μL CCK-8 reagent. After 2 h incubation, the well’s absorbance at 450 nm was measured by microplate reader. Each group set was repeated for five times and each measurement was performed three times repeatedly.

### Oil Red O Staining

Fresh or frozen tissues were fixed with 4% paraformaldehyde for 1 h at room temperature, and cell samples were washed with PBS and then fixed with 4% paraformaldehyde for 20min. Oil red O stain (Servicebio, G1015-100ML, Yikeshengwu, China) were operated in accordance with the manufacturer’s instructions.

### Western Blotting

Detailed steps of Western blotting are described (Zhou et al., 2010). Primary antibodies was NUSAP1 polyclonal antibody (ProteinTech, 12024-1-AP, United States) and GAPDH (ProteinTech Group, Rosemont, IL, United States) epitopes monoclonal antibodies. Then, the secondary antibody for Western blotting was the horseradish-peroxidase-labeled secondary antibody (ProteinTech Group, Rosemont, IL, United States).

### Statistical Analysis

All the contents of bioinformatics analysis were analyzed with R version 4.0.5 (https://www.r-project.org/). Student’s t-test was used to analyze the relative expression levels of hub genes. The *p* < 0.05 was considered statistically significant. *p < 0.05, **p < 0.01; ***p < 0.001; ns, not significant.

## Results

### Identification of DEGs Throughout NAFLD Progression

**FIGURE 1:**
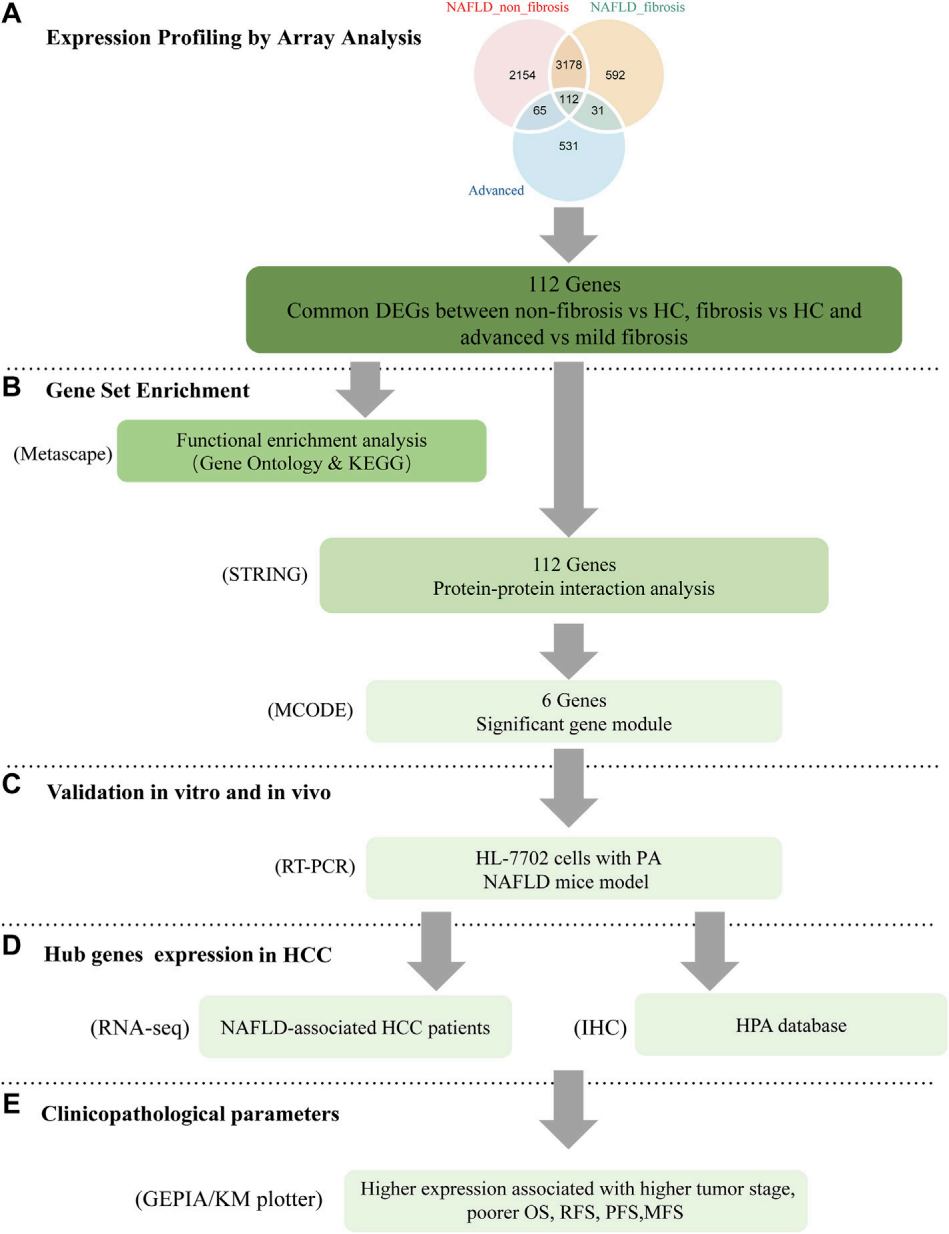
Workflow of the present study. **(A)** common DEGs between non-fibrosis vs HC, fibrosis vs HC and advanced vs mild fibrosis were identified by integrated bioinformatics analysis. **(B)** Enrichment analysis was carried by the Metascape online database. The hub genes were attained by PPI with STRING and MCODE with cytoscape. **(C)** The expression of key genes was verified by *in vitro* and *in vivo* experiments. The function of NUSAP1 was confirmed by silencing assay. **(D,E)** The expression of NUSAP1 in hepatocellular carcinoma and its relationship with prognosis were explored. DEGs, differentially expressed genes; HC, Healthy Control; FFA, free fatty acids; IHC, immunohistochemistry; HPA, The Human Protein Atlas.

**FIGURE 2:**
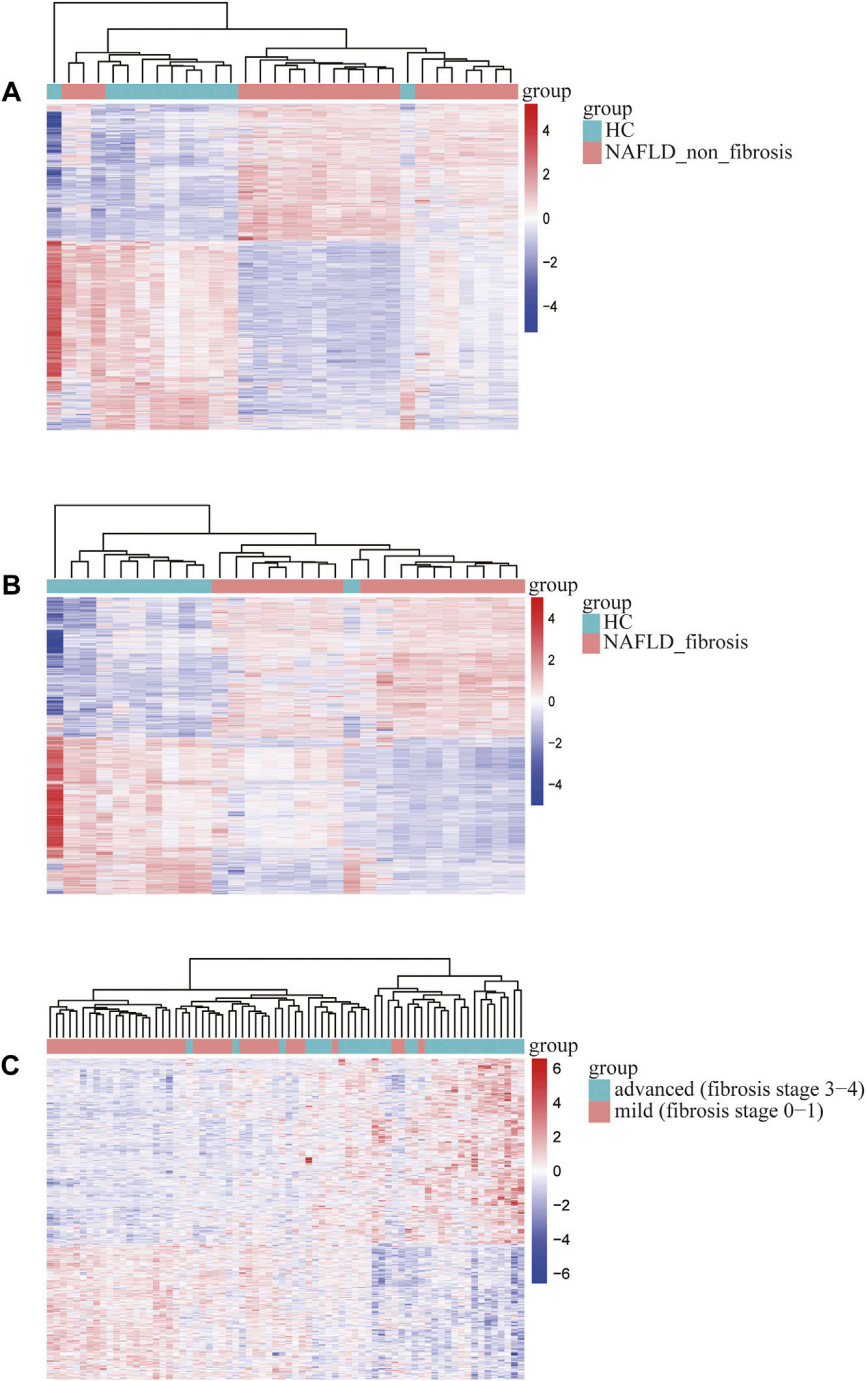
Heatmap of differentially expressed genes identified in **(A)** non-fibrosis vs. HC group in GSE89632, **(B)** fibrosis vs. HC group in GSE89632, **(C)** advanced vs. mild fibrosis in GSE49541. Legend on the top right indicates the relative expression levels of the genes.

The bioinformatics analysis workflow is summarized in Figure 1. DEGs related to fibrosis, and advanced fibrosis were identified by integrated bioinformatics analysis. We first determined the DEGs among different groups of GSE89632, which consisted of 18 cases with fibrosis, 21 cases with non-fibrosis and 11 healthy controls (HC). Total number of 5510 DEGs were determined for the non-fibrosis vs. HC group and a total of 3913 DEGs were identified from the fibrosis vs. HC group. Then, we analyzed the datasets GSE49541 (32 cases with advanced fibrosis and 40 cases with mild fibrosis) and identified 739 DEGs associated with advanced fibrosis. The list of differential genes in each group were visualized by the heatmaps whose hierarchical clustering was based on Euclidean distance (Figure 2)**.**

### GO and KEGG Pathway Analysis of Common DEGs

**FIGURE 3:**
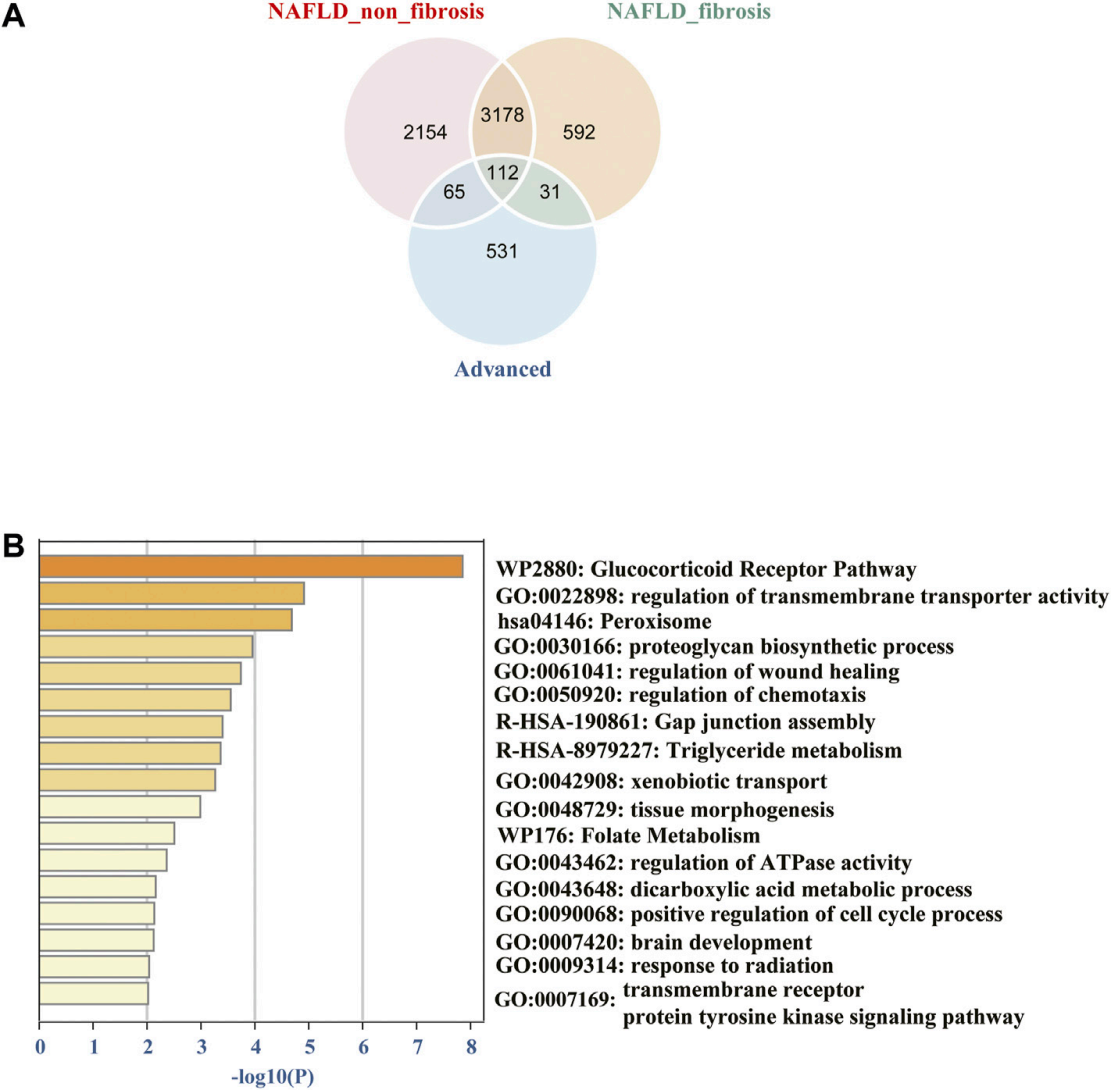
**(A)** Venn diagram of common differentially expressed genes from different groups among the two datasets. **(B)** The 112 common DEGs were carried out enrichment analysis by using Metascape. The brown column represents the–log10 (p-value) of the term. The result showed that the glucocorticoid receptor pathway, regulation of transmembrane transporter activity, peroxisome, proteoglycan biosynthetic process, regulation of wound healing, regulation of chemotaxis, etc. were the significantly enriched GO or pathways.

The common DEGs were showed using the “ggvenn” R package, which were significantly associated with progression of NAFLD from non-fibrosis to advanced fibrosis **(** Figure 3A). More specific information about the DEGs was shown in the additional file (Supplementary Table S2). To gain insight into biological significance of these genes, we carried out enrichment analysis by using the Metascape online database. The results showed that pathways such as glucocorticoid receptor pathway, regulation of transmembrane transporter activity, peroxisome, proteoglycan biosynthetic process, regulation of wound healing, regulation of chemotaxis, etc., were the significantly enriched GO or pathways (Figure 3B).

### PPI Network Analysis and Core Genes Selection

**FIGURE 4:**
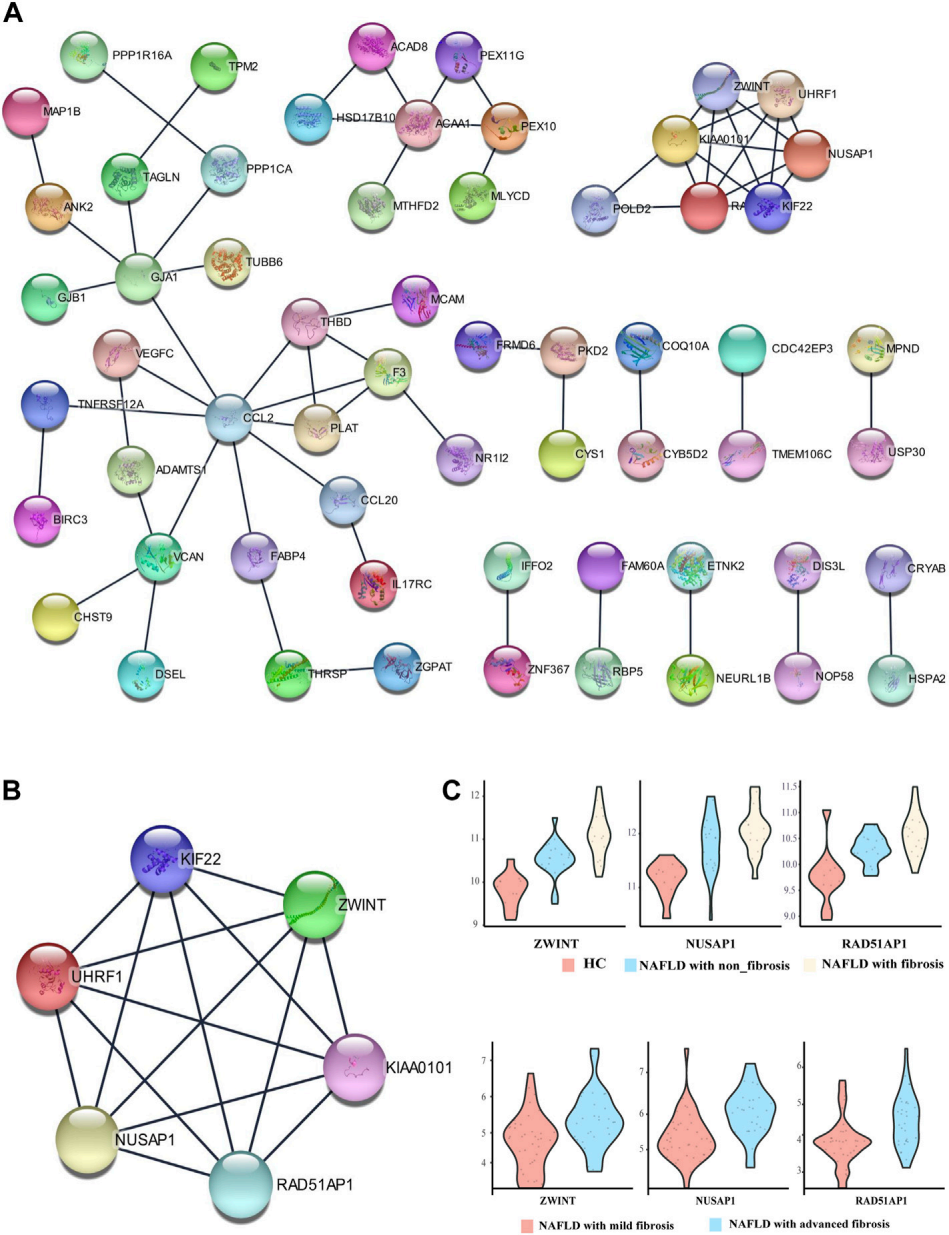
Protein-protein interaction (PPI) network. **(A)** PPI network of differentially expressed genes (DEGs), **(B)** subnetwork of top-six hub genes from the PPI network, **(C)** The expression pattern of ZWINT, NUSAP1 and RAD51AP1 in the different stage of NAFLD.

To uncover the potential relationship between 112 common DEGs, a PPI network that included 112 nodes and 65 edges were constructed using the STRING tool (Figure 4A). Then, the most significant modules were recognized by the MCODE plug-in of cytoscape. The top six hub genes in the MCODE cluster1 were selected (Table 2), including Kinesin Family Member 22 (KIF22), ZW10 interacting kinetochore protein (ZWINT), nucleolar and spindle associated protein 1 (NUSAP1), proliferating cell nuclear antigen-binding factor (KIAA0101), ubiquitin-like with PHD and ring finger domains 1 (UHRF1), RAD51-associated protein 1 (RAD51AP1) (Figure 4B). ZWINT, NUSAP1 and RAD51AP1 expression showed an increasing trend in the progression of NAFLD (Figure 4C **)**, but KIF22, KIAA0101 and UHRF1 exhibits a different expression profile in different stages of the NAFLD **(** Supplementary Figure S1 **)**.

**TABLE 2:** Hub genes with a high degree of connectivity.

Gene	MCODE Score	MCODE Cluster
KIF22	4.0	1
ZWINT	4.0	1
KIAA0101	3.73	1
UHRF1	3.73	1
NUSAP1	3.73	1
RAD51AP1	3.73	1

### Validation of Hub Genes Expression *in Vitro* and *Vivo*

**FIGURE 5:**
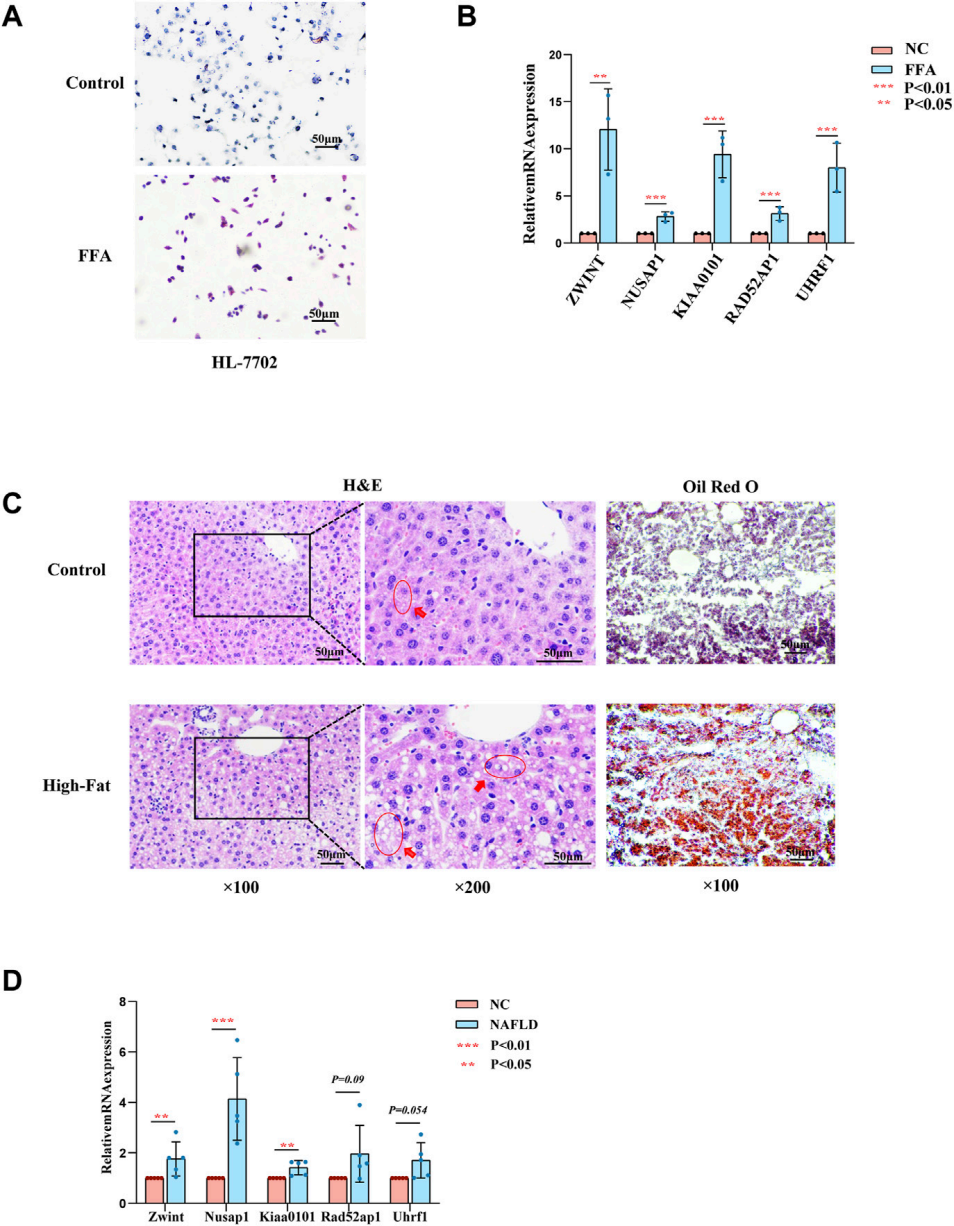
Experiments *in vitro* and *in vivo*. **(A)** Represent Oil Red O staining of HL-7702 cells treated with or without FFA for 24 h. **(B)** Expression levels of hub genes in HL-7702 cells treated with FFA for 24 h was assessed by RT-PCR (*n* = 3). **(C)** Represent HE (left, middle) and Oil Red O staining (right) of showing hepatic steatosis in liver tissues. 100ⅹfor the HE images (left), 200ⅹfor the HE images (midddle) and Oil red O images (right). **(D)** Expression levels of hub genes in NAFLD mice was assessed by RT-PCR (*n* = 3–5).

To further confirm the role of the hub genes in the progression of NAFLD, experimental validation was conducted *in vitro* and *vivo*. The expression of ZWINT, NUSAP1, RAD51AP1 were highly expressed in HL-7702 cells treated with FFA for 24 h as mentioned above by RT-PCR screening (Figures 5A,B). Due to a high homology between mouse and human genes, we also used mouse models of NAFLD established by HFD feeding for 12 weeks to further examine the hub gene expression levels. H&E and Oil red O staining of liver tissues exhibited compressed liver sinusoids, more dispersed lipid vacuoles and increased hepatocyte volumes in NAFLD mice than those in control mice (Figure 5C). The expression of *Zwint,* and *Nusap1* were significantly upregulated in NAFLD mice compared to the control group by RT-PCR, which was consistent with the results of our previous analysis. In contrast to our previous results, Kiaa0101 was significantly increased in NAFLD mouse. The expression levels of *Rad51ap1* and *Uhrf1* tended to increase in NAFLD mice but the difference was not statistically significant (*p* = 0.09 and *p* = 0.054) (Figure 5D). Therefore, NUSAP1 and ZWINT may be the key genes in the progression of NAFLD.

### NUSAP1 was Highly Expressed in NAFLD-Associated HCC was Associated With Cell Migration Cell Proliferation, Migration and Fat Accumulation Under High Fat Condition

**FIGURE 6:**
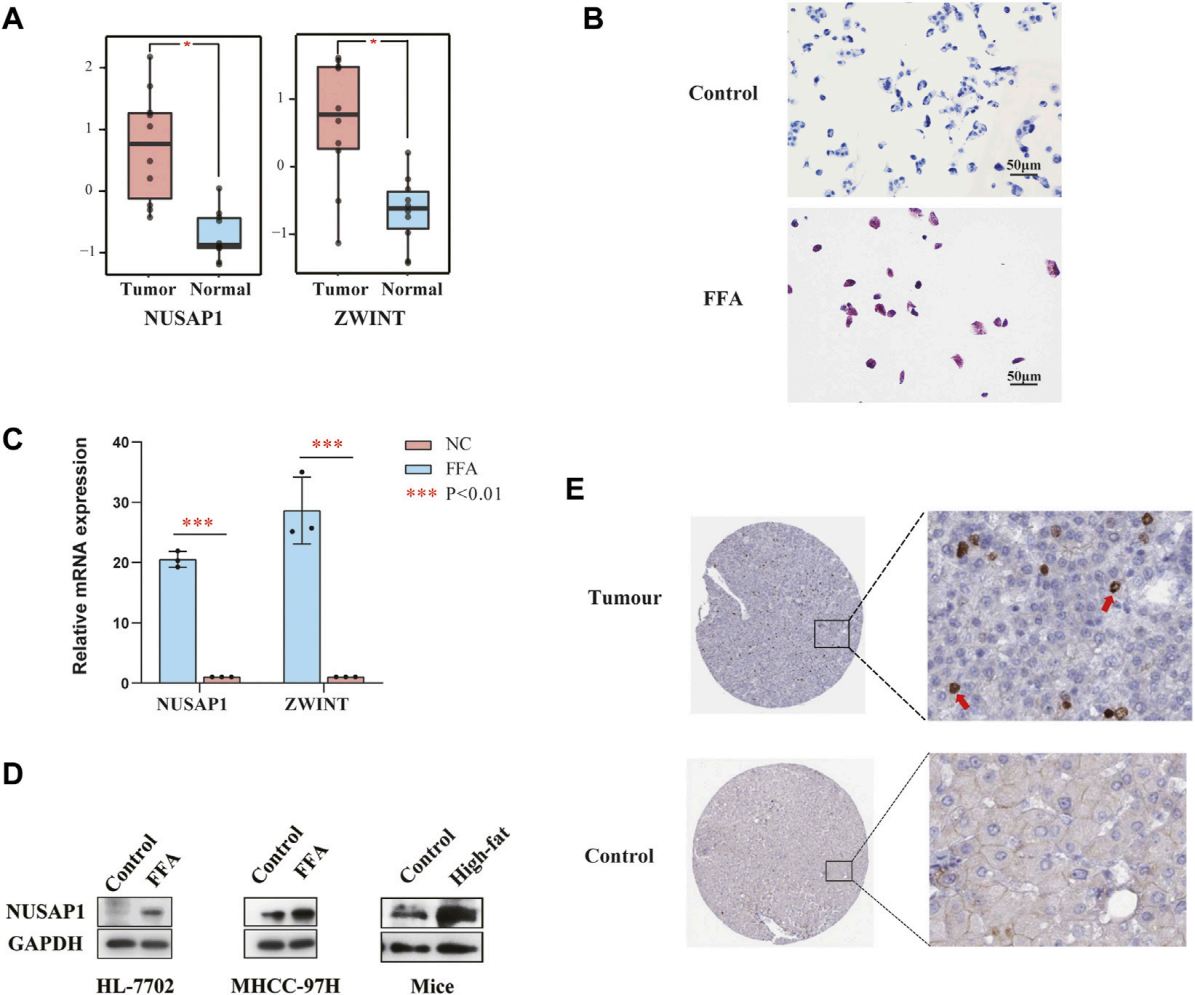
**(A)** Relative mRNA expression of NUSAP1 and ZWINT in NAFLD-related cancer tissues and paired paracancer tissue (***p < 0.001). **(B)** Represent Oil Red O staining of MHCC-97H cells treated with or without FFA for 24 h. **(C)** Expression levels of NUSAP1 and ZWINT in MHCC-97H cell lines treated with FFA for 24 h was assessed by RT-PCR (*n* = 3). The data were presented as mean ± SEM, *n* = 3 each group. *p < 0.05, ***p < 0.001. **(D)** The protein levels of NUSAP1 in HL-7702, MHCC-97H cell lines and NAFLD mice liver. *In vitro n* = 3 for each group, *in vivo*, *n* = 3–5 for each group. **(E)** The IHC-based protein expression of NUSAP1 in HCC tissues and normal liver tissues. All the IHC staining images were obtained from the HPA database (https://www.proteinatlas.org/ENSG00000137804-NUSAP1/pathology/liver+cancer#img).

**FIGURE 7:**
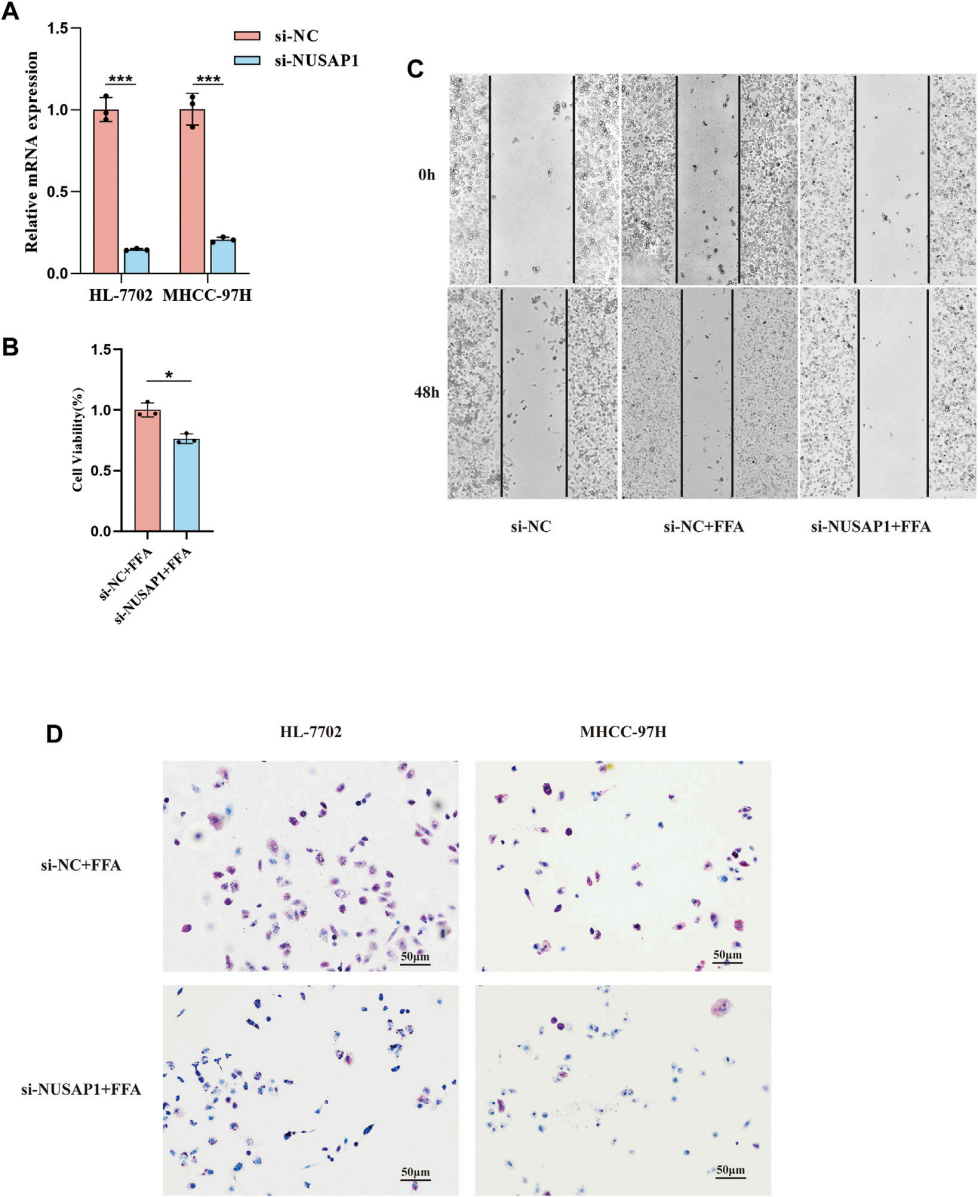
Validation of the function of NUSAP1 via the silencing assay. **(A)** Confirmation of NUSAP1 silence efficiency in HL-7702 and MHCC-97H cell lines by RT-PCR. **(B)** CCK-8 analysis of cell growth in MHCC97H (*n* = 3). **(C)** Wound-healing assays for MHCC-97H. **(D)** Oil Red O staining of the NUSAP1 silence in HL-7702 and MHCC-97H cell treated with FFA for 24 h, 100Xfor the images. The data were presented as mean ± SEM, *n* = 3 each group. *p < 0.05, ***p < 0.001.

To further inquire the expression of NUSAP1 and ZWINT in the (NAFLD)-associated HCC patients, we downloaded the data set GSE164441 including 10 paired NAFLD-associated HCC tumor and adjacent normal tissues. It was found that the expression of NUSAP1 and ZWINT was significant higher (*p < 0.05*) in tumours specimens compared with para-cancer tissues **(** Figure 6A). We then confirmed this result in *in vitro* cell line studies. NUSAP1 was highly expressed in MHCC-97H cell lines treated with 1 mmol/L of FFA for 24 h (Figures 6B,C). Then, we also investigated the protein expression of NUSAP1. Similarly, protein levels of NUSAP1 in liver normal cell HL-7702, liver cancer cell MHCC-97H and NAFLD mice liver significantly elevated (Figure 6D). What’s more, IHC results about the NUSAP1 expression, obtained from the HPA database, showed that NUSAP1 had a higher intensity in HCC tissues than that in normal liver tissues (Figure 6E). In order to further verify the functional characteristics of NUSAP1, we conducted the NUSAP1 silencing assay. We firstly confirmed the efficiency of si-NUSAP1 (Figure 7A). Then CCK-8 analysis and Wound healing assays indicated that knockdown of the NUSAP1 gene could result in a significant reduction in migration of MHCC-97H cell under high fat condition (Figures 7B,C). In addition, the lipid content of the cells reduced significantly after NUSAP1 silence (Figure 7D), suggesting that NUSAP1 may be associated with lipid **accumulation** in this study.

### NUSAP1 Correlated With Poorer Prognosis in Liver Cancer

**FIGURE 8:**
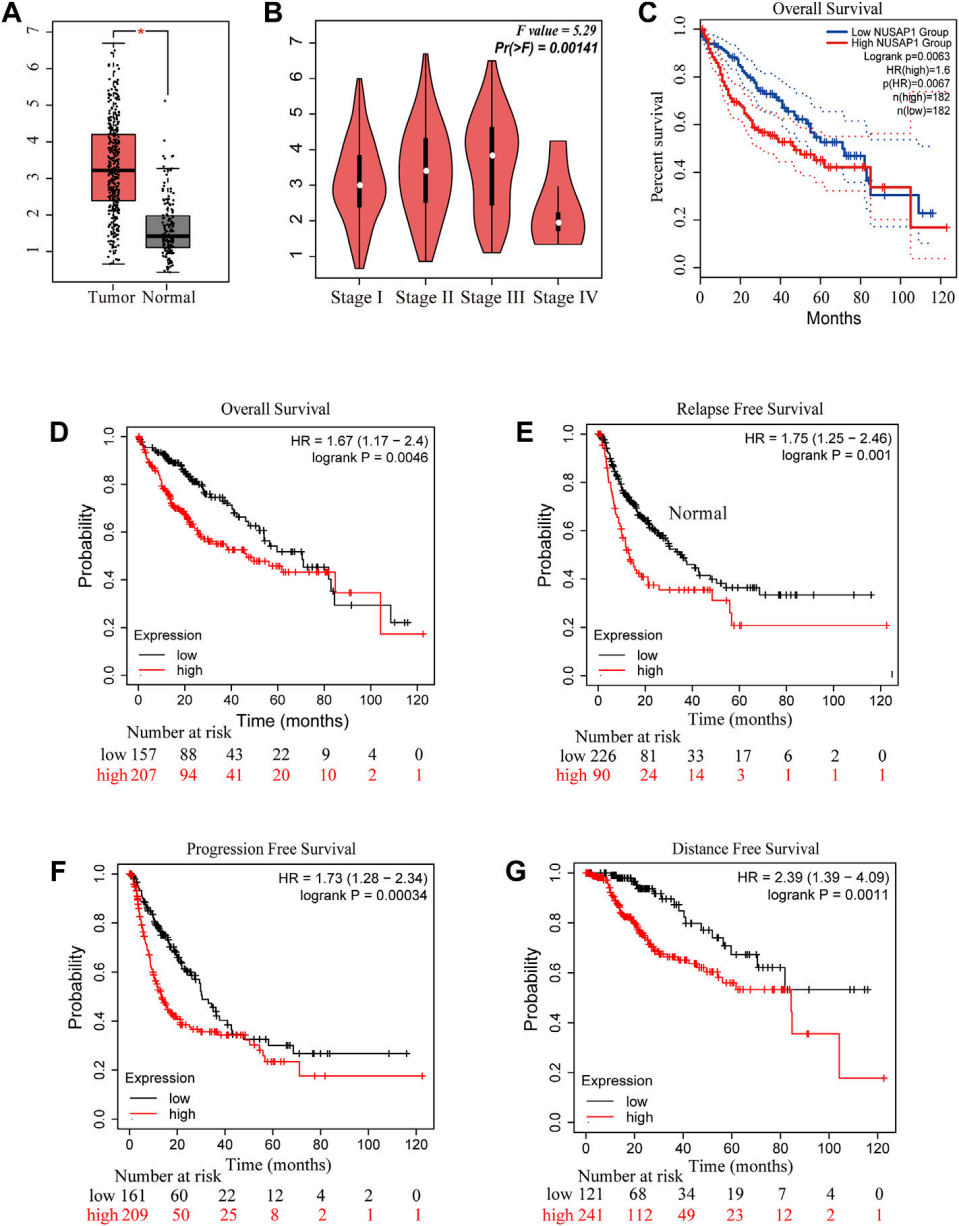
NUSAP1 was highly expressed in HCC and correlated with poorer prognosis**. (A)** The expression of NUSAP1 in liver cancer (GEPIA)**, (B)** Correlation between the expression of NUSAP1 and tumor stage in liver cancer patients (GEPIA). Correlation between the expression of NUSAP1 and OS **(C,D)**, RFS **(E)**, PFS **(F)** and DFS **(G)** in liver cancer patients [**(C)** from GEPIA: http://gepia.cancer-pku.cn/; **(D–G)** from KM plotter: http://kmplot.com/analysis/).

To further understand the role of NUSAP1 expression in HCC patients, the prognostic value of NUSAP1 was analyzed on the GEPIA and Kaplan-Meier Plotter website. The expression of NUSAP1 was significantly higher in liver cancer tissues than that in normal tissues (Figure 8A) and associated with tumor staging I-III but not stage IV (Figure 8B). In addition, the Kaplan-Meier curve and log-rank test analyses revealed that the increased NUSAP1 mRNA levels corresponded to the shortened OS (overall survival), RFS (relapse free survival), PFS (progression free survival) and DFS (distance free survival) in HCC (*p* < 0.05) (Figures 8C–G).

## Discussion

NAFLD is one of the most frequent metabolic diseases worldwide at present. It encompasses a wide spectrum of disorders including simple steatosis or nonalcoholic fatty liver (NAFL) and nonalcoholic steatohepatitis (NASH), which may progress to liver fibrosis, hepatic cirrhosis or even hepatocellular carcinoma (Pierantonelli and Svegliati-Baroni, 2019). A variable extent of fibrosis occurs in approximately 20–30% of patients with chronically NAFLD and the mortality rate attributable to NAFLD-related cirrhosis is around 12–25% (Massoud and Charlton, 2018). Another characteristic feature of NAFLD progression is HCC (Bessone et al., 2019). NAFLD-related cirrhosis accounts for 10–34% of the known causative factor of HCC, while patients with noncirrhotic NASH comprising 13–49% of all HCCs. At present, diabetes mellitus and obesity are considered as the major risk factors for the pathogenesis of NAFLD (Huang et al., 2021). The metabolic disorder of diabetes could promote the release of free fatty acids, which triggers the generation of pro-inflammatory cytokines and ROS (reactive oxygen species), and obesity could further stimulate the development of chronic liver inflammation. Hepatic accumulation of free fatty acids impairs mitochondrial functions and mediates death of CD4^+^ T lymphocytes selectively mediated by ROS, and reducing immune surveillance (Ma et al., 2016). In addition, it has been demonstrated that IgA^+^ cells accumulated in nonalcoholic steatohepatitis (NASH) and accelerated hepatocarcinogenesis via suppressing CD8^+^ T cells (Shalapour et al., 2017). Thus, the progression of NAFLD could contribute to the prevalence of cirrhosis and HCC worldwide. However, the molecular mechanisms on how NAFLD progresses from non-fibrosis to advanced fibrosis, which ultimately lead to HCC, are yet to be excavated.

In this current study, we extracted gene expression matrix of non-fibrosis NAFLD and fibrosis NAFLD tissue comparing to normal liver tissue or advanced fibrosis NAFLD tissue comparing to mild fibrosis from the two GEO datasets and screened out 112 co-expressed DEGs that could affect NAFLD progression. To further gain insight into the potential functions of these target genes, standard biological processes and pathways analysis of the common DEGs indicated that NAFLD progression from non-fibrosis to advanced fibrosis was inseparable from the glucocorticoid receptor pathway, regulation of transmembrane transporter activity, peroxisome and proteoglycan biosynthetic process. Studies has shown that the glucocorticoid receptor pathway was crucial for the progression of NAFLD. Glucocorticoid receptor β could induce hepatic steatosis via inflammation activation (Marino et al., 2016), and selective regulation on glucocorticoid receptor could reverse and prevent nonalcoholic fatty liver disease in mice models (Koorneef et al., 2018). Regulation of transmembrane transporter activity was proved to retard NAFLD progression by ameliorating hepatic glucose metabolic disorder (Guo et al., 2020). Peroxisome was also involved in multiple perturbed biological processes in NAFLD, including glucose and lipid metabolism, inflammation, and overall energy homeostasis (Francque et al., 2021). Peroxisome proliferator-activated receptors (PPARs) are a series of nuclear regulatory factors which regulate the activation of inflammatory cells and the fibrogenic process. It has been demonstrated that PPARs agonism shown favorable effect on liver morphology in phase IIb clinical trials (Ratziu et al., 2016; Francque et al., 2021). What is more, proteoglycan biosynthetic process was activated during the progression of NAFLD. For instance, TSK, a member of proteoglycan family that could link NAFLD to the development of atherogenic dyslipidemia and atherosclerosis (Mouchiroud et al., 2019). Therefore, we thought that these genes were strongly associated with NAFLD progression and worth further study.

Subsequently, 6 hub genes were identified following construction of the PPI network, which were considered as potential markers during the process of NAFLD progression. Through both *in vivo* and *in vitro* experiments, we demonstrated that ZWINT and NUSAP1 were up-regulated under high fat conditions and consistent with sequencing results. To identify genes linking NAFLD progression and HCC, we further explored the expression pattern and prognostic value of these two genes in NAFLD related HCC through public databases. NUSAP1 was not only up-regulated in NAFLD liver cancer but also associated with shorter OS (*p* < 0.05), RFS (*p* < 0.05), PFS (*p* < 0.05) and DFS (*p* < 0.05). In summary, NUSAP1 was identified as a target which could mediate NAFLD progression from no-fibrosis to advanced fibrosis which contributed to the NAFLD-HCC eventually.

NUSAP1 was a 55-kD nucleolar-spindle-associated protein expressed in proliferating cells preferentially (Raemaekers et al., 2003), which was involved in many biological processes such as mitosis, cytokinesis, kinetochore microtubule dynamics and chromosomal segregation (Li et al., 2016a; Li et al., 2016b). In addition, NUSAP1 also played essential roles in tumor progression. Michael et al. has demonstrated that NUSAP1 could serve as SCFcyclin F substrate that took part in the process of ubiquitin-dependent proteolysis during S and G2 phases of the cell cycle (Emanuele et al., 2011) and the presence of NUSAP1 was associated with more resistance formation upon chemotherapy agents (Emanuele et al., 2011). In liver cancer, knockdown of NUSAP1 could inhibit HCC cell proliferation, migration, and invasion *in vitro* and reduce its growth *in vivo* (Roy et al., 2018). In our study, we also demonstrated that NUSAP1 was high up-regulated in NAFLD patients and liver cancer cells under high fat conditions. NUSAP1 was also associated with cell proliferation and migration under high fat conditions and may promote lipid accumulation. That may be why NUSAP1 was elevated in NAFLD patients with liver fibrosis, where the capacity of regeneration of the liver by compensatory proliferation is dramatically reduced. More importantly, pro-inflammatory cytokines and ROS played an important role in the progress of NAFLD to HCC. Lipid accumulation results in chronic low-grade inflammation, triggering production of the cytokines IL-6 and TNF (Park et al., 2010), which induced cell proliferation and antiapoptotic pathways, and was found to be important for NASH-related HCC development (Kutlu et al., 2018). Therefore, cell proliferation might be an important NAFLD progression signatures, and NUSAP1 could serve as a prospective therapeutic target for NAFLD.

To conclude, 112 prospective candidate genes associated with NAFLD progression were screened out, which are involved in many pathways related to pathogenesis. All of these DEG should be further validated by experimental validation. Moreover, NUSAP1, ZWINT, KIAA0101, UHRF1, RAD51AP1, as prospective markers for NAFLD progression, they have not been linked previously, either in diagnosis or in research. Finally, we demonstrated that NUSAP1 had great potential as a drug target for preventing based on *in vitro* and *in vivo* experiments.

However, some limitations in this study also should be recognized. First of all, given the complexity of datasets included in this study, it is difficult to assess some confounding covariates when analyzing the DEGs, such as different ages, races, regions, and classification of patients. Secondly, the hub genes were all strongly linked to the disease progression of NAFLD, but the mechanism of regulation was not precise.

## Conclusion

Our study confirmed that the abnormally elevated expression of NUSAP1 was closely related to the progression of NAFLD patients from no-fibrosis to fibrosis, and even to HCC, indicating that their antagonism may inhibit or delay the process of NAFLD to HCC.

## Data Availability

The datasets presented in this study can be found in online repositories. The names of the repository/repositories and accession number(s) can be found in the article/Supplementary Material.

## Supplementary Material

Supplementary Figure S1 The expression pattern of UHRF1, KIF22 and ZWINT in the different stage of NAFLD.

## Abbreviations

DEGs, differentially expressed genes; DFS, distance free survival; GO, gene ontology; GEPIA2, gene expression profiling interactive analysis; HCC, hepatocellular carcinoma; KEGG, kyoto encyclopedia of genes and genomes; KIF22, kinesin family member 22; KIAA0101, proliferating cell nuclear antigen-binding factor; NUSAP1, nucleolar and spindle associated protein 1; NAFLD, non-alcoholic fatty liver disease; NASH, non-alcoholic steatohepatitis; OS, overall survival; PPI, protein-protein interaction; RAD51AP1, RAD51-associated protein 1; PPARs, peroxisome proliferator-activated receptors; RFS, relapse free survival; PFS, progression free survival; UHRF1, ubiquitin-like with PHD and ring finger domains 1; ZWINT, ZW10 interacting kinetochore protein.
