# Supplementary figures and images for: NUSAP1 Could be a Potential Target for Preventing NAFLD Progression to Liver Cancer
Source: Front Pharmacol. 2022 Apr 1;13:823140. doi: 10.3389/fphar.2022.823140 (PMC9010788; doi:10.3389/fphar.2022.823140)

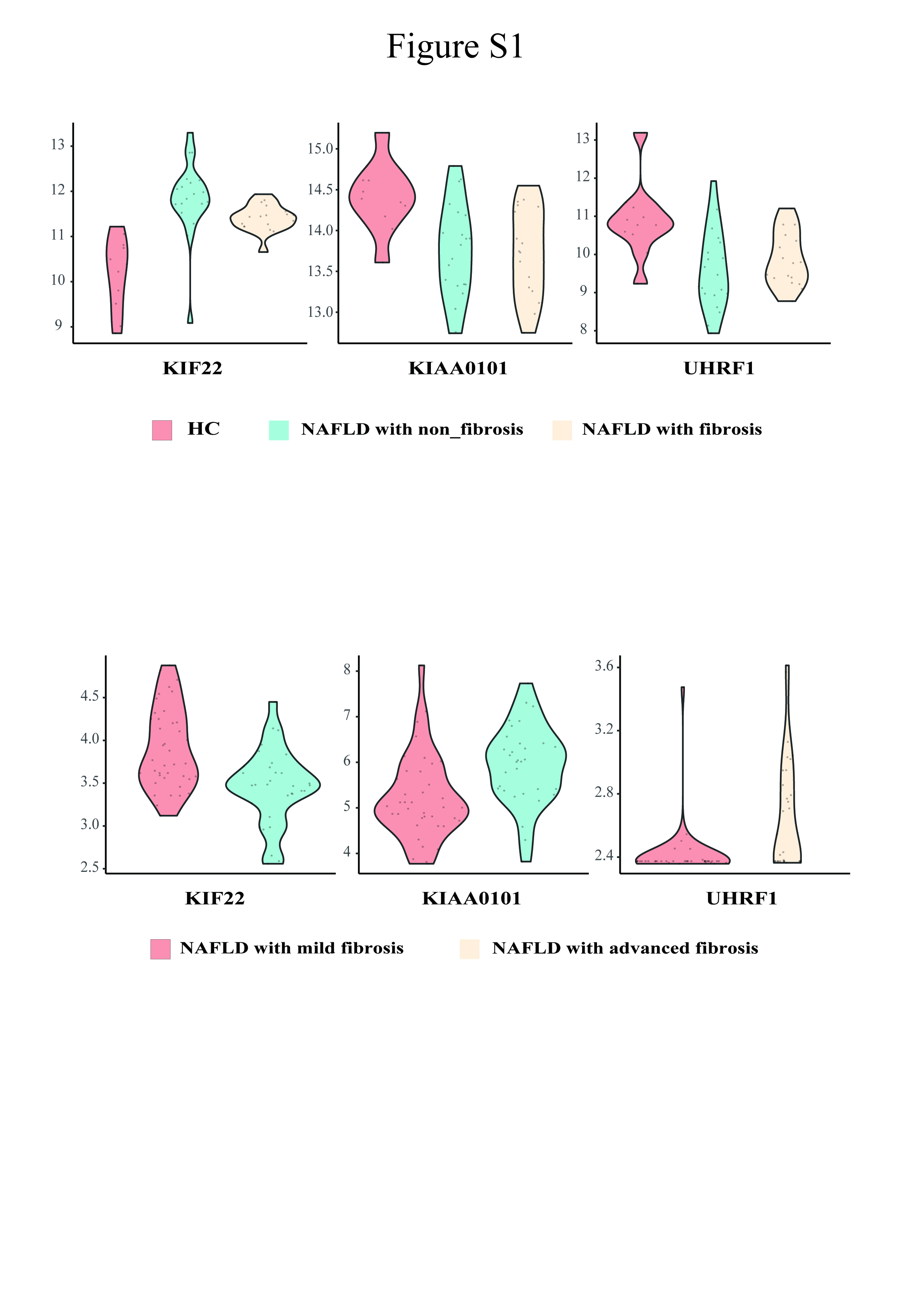

Supplement: Supplementary file 3 [file Image1.TIF]
